# Could Pan-Immune-Inflammation Value be a Marker for the Diagnosis of Coronary Slow Flow Phenomenon?

**DOI:** 10.1007/s12012-024-09855-4

**Published:** 2024-04-15

**Authors:** Mustafa Kaplangoray, Kenan Toprak, Edhem Deveci, Cuneyt Caglayan, Ebru Şahin

**Affiliations:** 1 Department of Cardiology, Faculty of Medicine, Bilecik Şehy Edebali University, Bilecik, Turkey; 2 Department of Cardiology, Faculty of Medicine, Harran University, Şanlıurfa, Turkey; 3 Department of Cardiology, University of Health Sciences, Mehmet Akif İnan Research and Training Hospital, Şanlıurfa, Turkey; 4 Department of Medical Biochemistry, Faculty of Medicine, Bilecik Şehy Edebali University, Bilecik, Turkey; 5 Department of Cardiology, Bilecik Training and Research Hospital, Bilecik, Turkey

**Keywords:** Coronary angiography, Coronary slow flow phenomenon, Inflammation, Pan-immune-inflammation value

## Abstract

Inflammation plays a key role in the pathogenesis of the coronary slow flow phenomenon (CSFP). The newly developed inflammatory marker, pan-immune-inflammation value (PIV), is associated with adverse cardiovascular events. This study investigated the predictive value of PIV for diagnosing CSFP in comparison to other inflammation-based markers. A total of 214 patients, 109 in the CSFP group and 105 in the normal coronary flow (NCF) group, were retrospectively included in the study. Coronary flow was calculated using the Thrombolysis in Myocardial Infarction frame count method. In addition to PIV, other inflammatory markers such as neutrophil–lymphocyte ratio, platelet-lymphocyte ratio (PLR), and systemic immune-inflammation index (SII) were calculated for the patients. The average age of patients was 50.3 ± 8.4, with a male ratio of 55.1%. Compared to the NCF group, patients in the CSFP group had higher levels of hyperlipidemia, glucose, triglyceride, NLR, PLR, SII, and PIV, while their high-density lipoprotein cholesterol (HDL-C), was lower (*p* < 0.05). Logistic regression analysis demonstrated that HDL-C, glucose, triglyceride, and PIV were independent predictor factors for CSFP (*p* < 0.05). PIV is a strong and independent predictor factor for CSFP and superior in predicting CSFP compared to other inflammatory markers.

## Introduction

Coronary slow flow phenomenon (CSFP) is an angiographic finding characterized by delayed opacification of the distal coronary artery without significant stenosis in the epicardial coronary arteries [1–3]. Its prevalence in patients undergoing coronary angiography due to suspected coronary artery disease ranges between 1 and 7% [4]. Studies have suggested that CSFP is related to inflammation, coronary anatomical structure, subclinical atherosclerosis, endothelial dysfunction, and small vessel disease, but its pathogenesis is yet to be understood in detail [5–11]. Inflammation is known to play a key role in cardiovascular diseases including atherosclerosis [12]. Recent studies also demonstrated that chronic inflammation-induced coronary microvascular dysfunction plays an important role in the pathophysiology of CSFP [13, 14].

Due to the association of inflammation with atherosclerosis, cardiovascular diseases, and heart failure, recent studies have focused on scoring systems based on haematological parameters and inflammatory markers [15]. The pan-immune-inflammation value (PIV), which reflects immune and inflammatory status and includes blood cells like neutrophils, lymphocytes, monocytes, and platelets, was initially shown to be an important prognostic marker in cancer patients [16]. Subsequent studies have demonstrated the relationship between the PIV with prognosis in ST-segment elevation myocardial infarction (STEMI) and heart failure. It has also been shown to be an independent factor for the development of no-reflow in patients undergoing primary percutaneous coronary intervention for STEMI [17, 18]. In addition, the neutrophil–lymphocyte ratio (NLR) and platelet-lymphocyte ratio (PLR), the systemic immune-inflammation index (SII), which are based on some of the parameters used in PIV and are associated with immune-inflammatory status, have been shown in many studies to be effective in predicting CSFP [13]. However, the relationship between CSFP and PIV have not been studied in detail. Therefore, this study was designed with the hypothesis that PIV could be a marker for the diagnosis of CSFP.

## Methods

### Study Population

This study was carried out between November 2022 and November 2023 at Bilecik Training and Research Hospital, Şanlıurfa Mehmet Akif İnan Training and Research Hospital and Harran University Faculty of Medicine. The angiographic records of 1825 patients who underwent coronary angiography (CAG) because of the presence of ischaemia on exercise electrocardiography or myocardial perfusion sinography, unstable angina pectoris, or cardiovascular risk factors and typical anginal symptoms were retrospectively reviewed. Those who did not have a significant stenotic lesion in their coronary arteries and whose coronary artery velocity was above normal values according to the Thrombolysis in Myocardial Infarction frame count (TFC) calculation, as reported in previous studies in any of the three coronary arteries, were determined as CSFP, and those with normal coronary anatomy and normal coronary velocity were defined as normal coronary flow (NCF) [19]. Accordingly, 109 patients in the CSFP group and 105 in the NCF group, totaling 214 patients, were included in the study.

Exclusion criteria were recent acute coronary syndrome, history of coronary revascularization, severe heart valve disease, congenital heart disease, decompensated heart failure, non-sinus rhythm, malignancy, severe liver and renal failure, acute or chronic infection, pulmonary disease, autoimmune disease, hematologic disease, anemia (hemoglobin below 12 g/dL for women and 13 g/dL for men as per World Health Organization criteria), any dilatation, spasm, and dissection in coronary arteries. The study was conducted by the Helsinki Declaration and was approved by the ethics committee of Bilecik Seyh Edebali University Faculty of Medicine. Retrospective consent was waived due to this being a retrospective study.

### Coronary Angiography

All patients underwent Judkins CAG via the femoral or radial route. The coronary flow velocities of the patients included in the study were measured by two experienced cardiologists using the Thrombolysis in Myocardial Infarction (TIMI) frame count (TFC) method [19]. The first frame number was determined when the proximal part of the coronary artery was more than 70% filled antegrade with contrast, and the last frame number was determined at the point where the contrast reached the mustache area for the left anterior descending artery (LAD), the distal bifurcation of the longest marginal optus branch for the circumflex artery (CX), and the first side branch to separate from the posterolateral artery for the right coronary artery (RCA). Since coronary angiography records in our units were measured at 15 frames per second, the frame numbers obtained for each vessel were multiplied by two. Since LAD is longer than the other two coronary arteries, the frame number obtained for LAD was divided by 1.7 to obtain the correct TFC (cTFC) value for LAD. TFC cut-off values were set as 21.1 ± 1.5 for LAD, 22.2 ± 4.1 for CX, and 20.4 ± 3 frames for RCA. The diagnosis of CSFP was made if the frame number in any of the coronary arteries exceeded the above-defined values. The intra- and interobserver variability values for TFC were calculated as 0.975 and 0.966, respectively. The mean TFC (mTFC) was calculated by dividing the sum of the TFC numbers obtained for LAD, CX, and RCA by three.

### Laboratory Measurements

Hematological, biochemical, and lipid parameters of patients were obtained from blood sample tests taken from the antecubital area after 12 h of fasting prior to CAG, as per hospital records. Using hematological test results,

$${\text{Neutrophil}} - {\text{Lymphocyte Ratio }}\left( {{\text{NLR}}} \right) \, = {\text{neutrophil count }}({1}0^{{3}} /\upmu {\text{L}})/{\text{lymphocyte count }}\left( {{1}0^{{3}} /\upmu {\text{L}}} \right),$$

$${\text{Platelet}} - {\text{Lymphocyte Ratio }}\left( {{\text{PLR}}} \right) \, = {\text{platelet count }}\left( {{1}0^{{3}} /\upmu {\text{L}}} \right)/{\text{lymphocyte count }}\left( {{1}0^{{3}} /\upmu {\text{L}}} \right),$$

$${\text{Systemic Immune}} - {\text{Inflammatory Index }}\left( {{\text{SII}}} \right) = {\text{ platelet count }}\left( {{1}0^{{3}} /\upmu {\text{L}}} \right) \times {\text{neutrophil count }}\left( {{1}0^{{3}} /\upmu {\text{L}}} \right)/{\text{lymphocyte count }}\left( {{1}0^{{3}} /\upmu {\text{L}}} \right),$$

$${\text{Pan}} - {\text{Immune}} - {\text{Inflammation value }}\left( {{\text{PIV}}} \right) \, = {\text{ neutrophil count }}\left( {{1}0^{{3}} /\upmu {\text{L}}} \right) \times {\text{platelet count }}\left( {{1}0^{{3}} /\upmu {\text{L}}} \right) \times {\text{monocyte count }}\left( {{1}0^{{3}} /\upmu {\text{L}}} \right)/{\text{lymphocyte count }}\left( {{1}0^{{3}} /\upmu {\text{L}}} \right)$$

were calculated [18].

### Comorbidity Definition

Patient medical records were carefully reviewed for comorbidity status and medication history. Diabetes Mellitus (DM) was defined as a fasting glucose level > 126 mg/dL, hemoglobin A1c (HbA1c) > 6.5%, or use of antidiabetic drugs. Dyslipidemia was diagnosed based on one of the following four criteria obtained from blood sample analysis after 12 h of fasting: (1) total cholesterol > 200 mg/dL, (2) low-density lipoprotein cholesterol (LDL-C) > 130 mg/dL, (3) high-density lipoprotein cholesterol (HDL-C) < 40 mg/dL in men and < 50 mg/dL in women, and triglyceride level > 150 mg/dL. The body mass index of the patients was calculated by dividing the weight by the square of the height (m^2^).

### Statistical Analysis

Statistical Package for the Social Sciences (SPSS for Windows, version 22.0, IBM Corp., Armonk, NY, U.S., 2016) was used for statistical analysis. Continuous variables with normal distribution were presented as mean ± standard deviation, and those not normally distributed were presented as median and interquartile ranges. Categorical variables were presented as percentages and compared using the Chi-Square test. Kolmogorov–Smirnov test was used to assess data normality. For comparing groups, independent-samples *t* test for normally distributed continuous variables and Mann–Whitney *U* test for non-normally distributed parameters. Spearman Rho Correlation Coefficient analysis was used to determine the correlation between PIV and TFC. Receiver operating characteristic (ROC) curve analysis was used to determine the PIV cut-off value predicting CSFP. The performance of PIV and other inflammation-based markers in diagnosis was assessed using areas under the ROC curve. Multivariate regression analyses were performed to determine the independent predictors of presence of CSFP. Baseline variables with significant significance (*p* < 0.05) by univariate analysis were included in the multivariate logistic regression analysis. In addition, the discriminative values of PIV and other inflammation-based markers on CSFP were attempted to be determined using Medcalc version 19.6.4 statistical software (MedCalc Software Ltd, Ostend, Belgium) by making pairwise comparison of the areas under the ROC curves with the DeLong test. The odds ratios (ORs) were presented with 95% respective confidence intervals (CI). A p-value of less than 0.05 was considered statistically significant.

## Results

The baseline characteristics and laboratory parameters of the patients included in the study are shown in Tables 1 and 2. Except for hyperlipidemia, both groups were similar in baseline characteristics (*p* > 0.05); however, the prevalence of hyperlipidemic patients was higher in the CSFP group (47.7% vs. 39.0%, *p* = 0.041). When comparing laboratory and angiographic findings between the two groups, patients in the CSFP group was observed to have higher levels of glucose, triglyceride, monocyte, neutrophil, NLR, PLR, SII, and TFC, while HDL-C and lymphocyte counts were lower (*p* < 0.05) (Table 2).

**Table 1:** Baseline characteristics of the two groups

	CSFP group (*n* = 109)	NCF group (*n* = 105)	*P* value
Age, years	50.98 ± 8.2	49.69 ± 8.5	0.259
Male sex, *n* (%)	65, (59.6)	53, (50.5)	0.178
BMI, kg/m^2^	26.3 ± 4.1	25.4 ± 4.7	0.101
LVEF %	60 (59.5–62.0)	61 (60.0–64.0)	0.135
Smoking, *n* (%)	46, (42.2)	42, (40.1)	0.357
Hyperlipidemia, *n* (%)	52, (47.7)	39, (39.0)	0.041
Hypertension, *n* (%)	45, (41.3)	42, (40.0)	0.848
Diabetes mellitus, *n* (%)	46, (42.2)	38, (36.2)	0.402
CCB, *n* (%)	18, (16.5)	9, (8.6)	0.080
Beta-blocker, *n* (%)	27, (24.8)	20, (19.0)	0.312
ACEI/ARB, *n* (%)	31, (28.4)	30, (28.8)	0.948
Antiplatelet, *n* (%)	42, (38.5)	34, (32.4)	0.347
Statin, *n* (%)	28, (25.7)	19, (18.1)	0.120

*ACEI* angiotensin-converting enzyme inhibitor, *ARB* angiotensin II receptor blocker, *CSFP* coronary slow flow phenomenon, *CCB* **c**alcium channel blockers, *NCF* normal coronary flow

**Table 2:** Biochemical and angiographic findings of the two groups

	CSFP group (*n* = 109)	NCF group (*n* = 105)	*P* value
Glucose (mg/dL)	110 (98.0–144.5)	102 (93.0–115.5)	0.003
Urea (mg/dL)	30 (24.0–35.0)	28.5 (23.1–37.0)	0.645
Creatinine (mg/dL)	0.84 (0.76–1.0)	0.82 (0.71–0.94)	0.102
LDL-C (mg/dL)	125 (105.8–142.0)	122 (98.0–145.0)	0.264
HDL-C (mg/dL)	36.6 (33.0–41.0)	41 (36.9–45.0)	0.012
Total cholestero, (mg/dL)	187 (171.6–212)	180 (167–208)	0.082
Triglyceride (mg/dL)	172 (150.5–188.0)	152 (130.8–175.0)	< 0.001
Hemoglobin (gr/dL)	14.1 (13.2–16.0)	13.4 (12.9–15.0)	0.101
White blood cell count (10^3^/µL)	8.1 (7.2–9.3)	7.7 (6.2–8.9)	0.056
Platelets (10^3^/µL)	243 (2160–283)	230 (200–279)	0.287
Lymphocyte (10^3^/µL)	1,8. (1.5–2.5)	2.1 (1.7–2.7)	0.045
Monocyte (10^3^/µL)	0.9 (0.7–1.2)	0.79 (0.6–1.0)	0.003
Neutrophil (10^3^/µL)	3.8 (3.0–5.6)	3.1 (2.4–3.8)	< 0.001
NLR	1.9 (1.4–2.5)	1.5 (1.2–1.9)	0.010
PLR	136 (111.5–162.0)	126 (101–146)	0.020
SII	453 (336–587)	339 (245–472)	0.001
PIV	443 (243–557)	261 (186–377)	< 0.001
TIMI frame count			
LAD (corrected)	41.5 (36.5–45.6)	18.5 (13.0–20.1)	< 0.001
LCX	28.4 (24.3–31.6)	15.0 (12.0–20.0)	< 0.001
RCA	32.2 (26.5–39.7)	18.0 (13.0–20.0)	< 0.001
Mean	33.0 (29.6–36.2)	17.0 (14.4–19.3)	< 0.001
Distribution of CSFP among major coronary arteries			
LAD, *n* (%)	61 (55.9)		
LCX, *n* (%)	49 (44.9)		
RCA, *n* (%)	55 (50.4)		
Number of coronary arteries involved			
1, *n* (%)	49 (44.9)		
2, *n* (%)	33 (30.2)		
3, *n* (%)	27 (24.7)		

*CSFP* coronary slow flow phenomenon, *LDL* low-density lipoprotein cholesterol, *HDL* high-density lipoprotein cholesterol, *LAD* Left anterior descending artery, *LCX* circumflex artery, *RCA* right coronary artery, *TIMI* thrombolysis in myocardial infarction, *NCF* normal coronary flow, *NLR* neutrophil-to-lymphocyte ratio, *PLR* platelet-to-lymphocyte ratio, *SII* systemic immune-inflammation index, *PIV* pan-immune-inflammation value

**Fig. 1:**
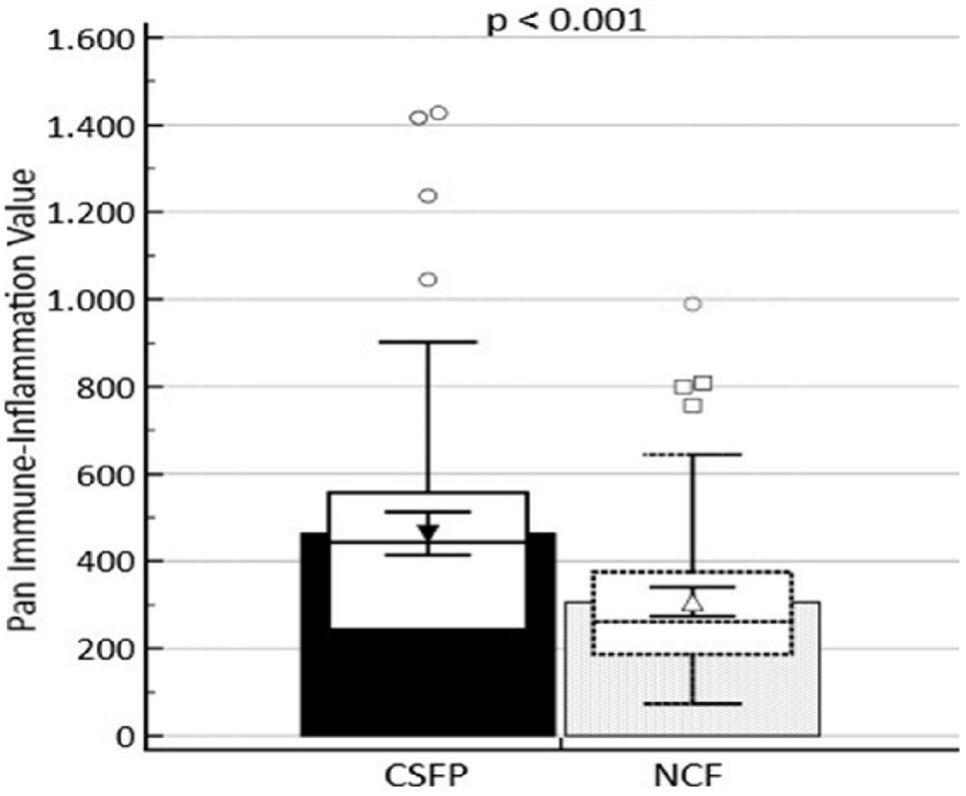
PIV according to the presence or absence of coronary slow flow phenomenon (*CSFP* coronary slow flow phenomenon, *NCF* normal coronary flow)

**Fig. 2:**
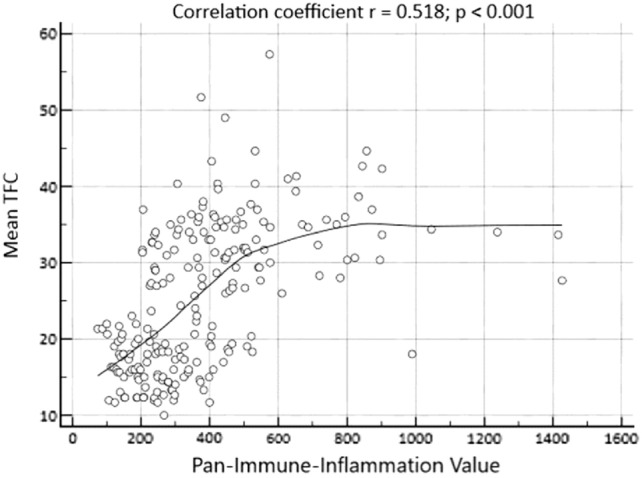
Correlation analysis between PIV and the mean thrombolysis in myocardial infarction frame count (TFC)

The PIV of patients in the CSFP group was observed to be higher compared to the NCF group (*p* < 0.001) (Fig. 1). Spearman Rho Correlation Coefficient analysis revealed a significant positive correlation between PIV and mean TFC (*r* = 0.518, *p* < 0.001) (Fig. 2).

Multivariate logistic regression analysis identified glucose, triglyceride, HDL-C, and PIV as independent and significant factors for CSFP (Table 3).

**Table 3:** Univariate and multivariate logistic regression analysis for presence of CSFP

Variables	Univariate regression analysis		Multivariate regression analysis	
	Odds ratio (95% CI)	*P* value	Odds ratio (95% CI)	*P* value
Hyperlipidemia	1.041 (0.694–1.142)	0.031	0.322 (0.133–1.018)	0.083
Glucose	0.989 (0.982–0.996)	0.003	1.012 (1.001–1.022)	0.035
Triglyceride	0.985 (0.978–0.993)	< 0.001	1.017 (1.005–1.029)	0.005
HDL-C	1.050 (1.006–1.096)	0.025	0.909 (0.871–0.948)	< 0.001
NLR	0.629 (0.417–0.852)	0.010	0.728 (0.361–1.468)	0.375
PLR	0.994 (0.989–0.999)	0.011	1.000 (0.992–1.008)	0.983
SII	0.998 (0.996–0.999)	0.003	1.001 (0.998–1.003)	0.550
PIV	0.996 (0.995–0.999)	< 0.001	1.012 (1.008–1.015)	< 0.001

*CSFP* Coronary slow flow phenomenon, *WBC* white blood cell count, *NLR* neutrophil-to-lymphocyte ratio, *PLR* platelet-to-lymphocyte ratio, *SII* systemic immune-inflammation index, *PIV* pan-immune-inflammation value, *HDL-C* high-density lipoprotein cholesterol

**Fig. 3:**
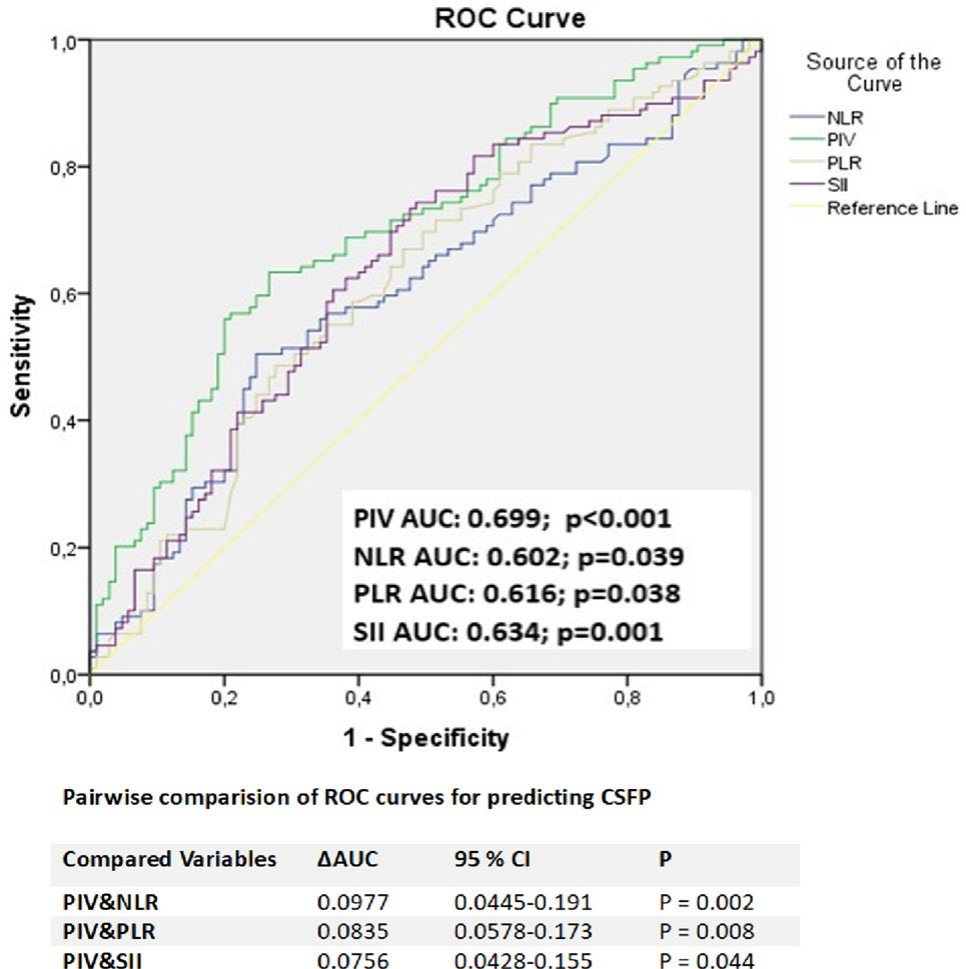
Comparison of ROC curves of PIV, SII, PLR, and NLR for predicting CSFP. At the best cut-off value of 338.1, PIV predicted CSFP with 64.2% sensitivity and 61.9% specificity, and the power of PIV to predict poor CSFP was superior to NLR, PLR, and SII

ROC curve analysis determined the optimal PIV cut-off value for predicting CSFP diagnosis as 338.1 (sensitivity 64.2%, specificity 61.9%, area under the receiver operating characteristic curve, 0.699; *p* < 0.001). ROC curve analysis results showed that PIV’s discriminative capacity in predicting CSFP was superior to all other indexes when comparing the AUC values for PIV, NLR, PLR, and SII (Fig. 3).

## Discussion

This study demonstrated that glucose, triglyceride, HDL-C, and PIV are independent predictors for CSFP. In addition, this study showed that PIV had a higher predictive value for CSFP than other inflammation-based markers, NLR, PLR and SII. To the best of our knowledge, this is the first study to directly compare the predictive strength of inflammation-based markers for CSFP.

Although the pathophysiological mechanism of CSFP, a common angiographic finding encountered by interventional cardiologists, is not fully understood, there is substantial amount of data supporting the relationship between CSFP and microvascular dysfunction. Potential mechanisms thought to be involved in the development of CSFP include vasoactive autacoids, diffuse atherosclerosis, abnormal platelet functions, and endothelial dysfunction [4]. All of these factors could contribute to the microvascular coronary dysfunction and the development of CSFP, which requires further research to clarify. In addition, several studies have established the relationship between inflammation and CSFP [20]. Studies on the role of inflammation in CSFP have shown that indexes based on more than one marker, rather than a single inflammation-related marker, have more power to predict CSFP [18, 21–23]. The results of our study also support this theory.

This study investigated the relationship between PIV, an immune-inflammation-based marker obtained from peripheral blood comprising four types of blood cells, and CSFP. Consistent with previous studies, patients in the CSFP group were found to have higher levels of PIV, NLR, PLR and SII compared to the NCF group. Logistic regression analysis revealed that only PIV was an independent predictor factor for CSFP. Additionally, pairwise analysis demonstrated that PIV's predictive effect for CSFP was stronger than NLR, PLR, and SII. Due to its comprehensive properties, PIV has become widely recognized as an inflammatory marker for various oncological diseases [24, 25]. Later, the prognostic role of PIV in other inflammatory diseases such as sepsis was demonstrated [26]. Besides chronic inflammation, the use of PIV in cardiovascular disorders is also biologically plausible [27]. As it is known, NLR and PLR include two parameters, SII includes three parameters, while PIV includes four parameters: neutrophils, platelets, monocytes and lymphocytes. Both the adhesion of platelets and the secretion of procoagulant substances by platelets play important roles in the development and progression of coronary artery disease. Similar to platelets, the neutrophils and monocytes are crucial in atherosclerosis [28]. In particular, neutrophils contribute to the formation of all atherosclerotic plaque processes, both directly by invading the plaque and indirectly through the proteolytic enzymes and arachidonic acid they release [29]. Considering the contributions of peripheral blood cells to coronary microanatomy, the division of monocytes, neutrophils, and platelets into lymphocytes in the PIV score has a strong biological rationale. A recent study analyzed the predictive effectiveness of preoperative PIV and reported that it was superior to NLR, PLR, and SII in predicting in-hospital and long-term mortality in STEMI patients [17].

The current study also showed that glucose, HDL-C, and triglyceride levels are independent predictor factors for CSFP. These findings are in parallel with the findings of Afşin et al. [30]. In their study, they demonstrated that the atherogenic plasma index (AIP), also known as the ratio of triglyceride to HDL-C, is an independent factor for CSFP. AIP is considered to be an indirect indicator of small dense LDL-C. Small dense LDL-C is known to play a significant role in the development of atherosclerosis, and diffuse atherosclerosis is a suspected key parameter in CSFP pathogenesis [31]. The results of current study support the role of diffuse atherosclerosis in CSFP pathogenesis. Consistent with previous studies showing an association between low HDL-C and high TG levels and CSFP [32, 33], we found low HDL-C levels along with high TG levels in the CSFP group in our study.

Another important finding of this study is that hyperglycemia is a predictor factor for CSFP. Hyperglycemia can occur due to increased levels of stress hormones such as steroids, catecholamines, glucagon, and decreased insulin levels due to stress [34]. It is thought that hyperglycemia-induced free radicals contribute to microvascular endothelial dysfunction and inflammation, leading to CSFP. This is thought to cause CSFP by causing microvascular endothelial dysfunction and inflammation due to increased free radicals as a result of hyperglycemia [35, 36]. Several different studies have reported endothelial dysfunction caused by high glucose concentrations in the peripheral arteries of patients with impaired glucose tolerance [36, 37]. A study by Binak et al. reported that impaired glucose tolerance may be an independent etiological factor for CSFP [38]. Our results are consistent with the findings of a study by Xia et al., which showed that 2-h postprandial glucose levels are an important factor for the development of CSFP [39].

### Limitations

This study has some limitations. In particular, the retrospective nature and the relatively small number of patients with CSFP limited the analyses. Other parameters that may be associated with inflammation have not been investigated within the scope of this study. A more comprehensive inflammation panel, including cytokines, and similar biomarkers, could provide further insights. If the results of current study are supported by prospective evidence, the predictive value of PIV in the diagnosis of CSFP is expected to increase in the future.

## Conclusion

The results of this study support that the increased value of PIV, an easily accessible and inexpensive biomarker, is an important and independent parameter for CSFP. Another important result of the study was that the power of PIV to predict the diagnosis of CSFP was stronger than other inflammatory-based markers. More comprehensive studies are needed before this parameter can be used routinely in the diagnosis of CSFP.

## Data Availability

All data generated or analyzed during this study are included in this published article.
